# Machine learning for prediction of delirium in patients with extensive burns after surgery

**DOI:** 10.1111/cns.14237

**Published:** 2023-04-30

**Authors:** Yujie Ren, Yu Zhang, Jianhua Zhan, Junfeng Sun, Jinhua Luo, Wenqiang Liao, Xing Cheng

**Affiliations:** ^1^ Medical Center of Burn Plastic and Wound Repair The First Affiliated Hospital of Nanchang University Nanchang China; ^2^ Medical Innovation Center The First Affiliated Hospital of Nanchang University Nanchang China; ^3^ Medical Center of Burns and Plastic Ganzhou People's Hospital Ganzhou China

**Keywords:** delirium, extensive burns, external validation, machine learning, prediction model

## Abstract

**Aims:**

Machine learning‐based identification of key variables and prediction of postoperative delirium in patients with extensive burns.

**Methods:**

Five hundred and eighteen patients with extensive burns who underwent surgery were included and randomly divided into a training set, a validation set, and a testing set. Multifactorial logistic regression analysis was used to screen for significant variables. Nine prediction models were constructed in the training and validation sets (80% of dataset). The testing set (20% of dataset) was used to further evaluate the model. The area under the receiver operating curve (AUROC) was used to compare model performance. SHapley Additive exPlanations (SHAP) was used to interpret the best one and to externally validate it in another large tertiary hospital.

**Results:**

Seven variables were used in the development of nine prediction models: physical restraint, diabetes, sex, preoperative hemoglobin, acute physiological and chronic health assessment, time in the Burn Intensive Care Unit and total body surface area. Random Forest (RF) outperformed the other eight models in terms of predictive performance (ROC:84.00%) When external validation was performed, RF performed well (accuracy: 77.12%, sensitivity: 67.74% and specificity: 80.46%).

**Conclusion:**

The first machine learning‐based delirium prediction model for patients with extensive burns was successfully developed and validated. High‐risk patients for delirium can be effectively identified and targeted interventions can be made to reduce the incidence of delirium.

## Graphical abstract

Developed and validated the first delirium prediction model for patients with extensive burns. The predictive model can effectively identify high‐risk patients for intervention, reduce the incidence of delirium, alleviate the physical, psychological, and financial burden on patients, and rationalize the allocation of healthcare resources.

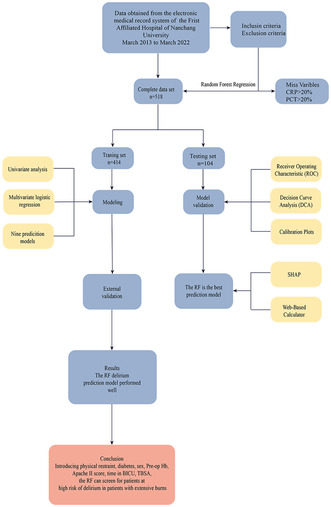

## 1 INTRODUCTION

Burns are an under‐recognized trauma and the number of burn cases worldwide is still on the rise, with a very high mortality rate. Especially with extensive burns, the injury leads to damage to the skin barrier and bacterial invasion, causing systemic sepsis and septicemia, and also predisposes to multi‐organ dysfunction.^1^ The treatment of extensive burns is lengthy and complex, with various complications, one of which is delirium,^2^ which incidence can be as high as 26%–77%.^3^, ^4^ Delirium is an acute brain dysfunction, a clinical syndrome caused by various causes, with impairment of attention, consciousness and cognition.^5^ Delirium exacerbates the patient's condition and leads to longer hospital stays,^6^ increased mortality,^7^ higher hospital costs,^8^ cognitive decline and even dementia in patients with extensive burns.^9^, ^10^ Despite this, there are very few studies on delirium in combination with extensive burns and a lack of clear efficacy in the pharmacological treatment of delirium.^11^, ^12^ It is worth noting that there is evidence that aggressive interventions are effective in reducing the risk of delirium during hospitalization in high‐risk patients.^13^

Machine learning is widely used in clinical settings and does not have a universally agreed definition,^14^ but is generally considered to be the process of identifying groups of information in data.^15^ Currently, machine learning models are widely used in medical fields, such as data mining, medical diagnosis, and disease risk prediction,^16^ which have good predictive performance.^17^ Risk factors for delirium have been used to develop predictive models for delirium in different populations, but no predictive models for delirium in patients with extensive burns have been developed.^18^, ^19^ It is difficult to achieve cross‐population usage between existing predictive models.^18^ Therefore, by studying the risk factors of combined delirium in patients with extensive burns, we established a machine learning‐based delirium prediction model, visualized the model and further promoted it to the clinic, aiming to help healthcare professionals identify high‐risk patients early, reduce the incidence of delirium in patients with extensive burns, and improve the prognosis of patients.

## 2 MATERIALS AND METHODS

### 2.1 Study subjects, design and data collection

This study complies with the Declaration of Helsinki and has been approved by the institutional ethics committee, certification (2022)CDYFYYLK(09‐052). Since this study is an observational study and no interventions were administered to the patients, written consent of the patients and their families was waived. Patients with large burns who met the inclusion and exclusion criteria and were admitted to the burn unit of the First Affiliated Hospital of Nanchang University from March 2013 to March 2022 were selected for this study. The data collected from the First Affiliated Hospital of Nanchang University was used to develop the prediction model. We externally validated the best prediction model using data on patients with large burns admitted from another large tertiary hospitals, the Ganzhou People's Hospital, from September 2017 to September 2022.

**FIGURE 1:**
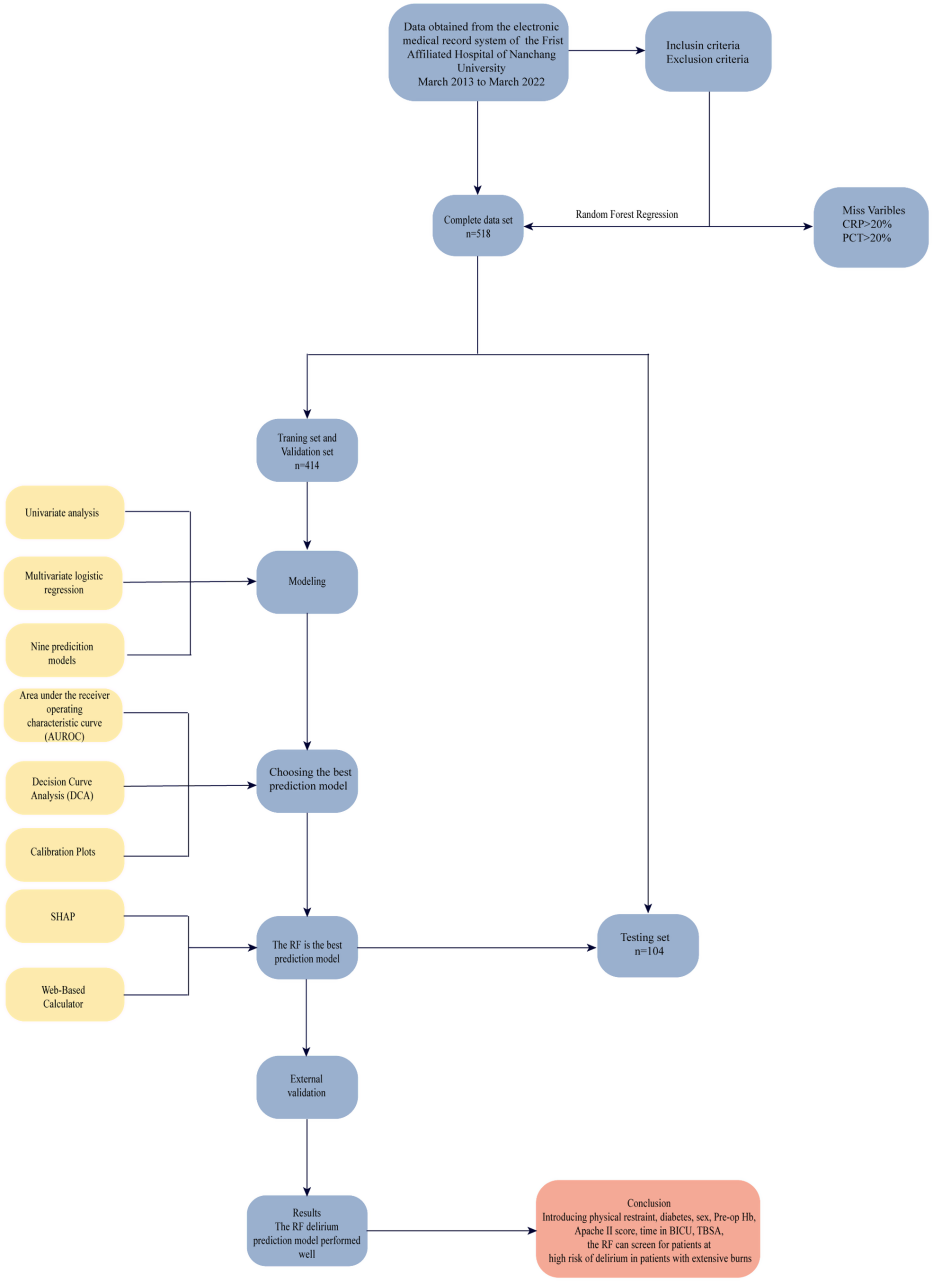
Flow chart of study design route. APACHE II, Acute Physiology and Chronic Health Evaluation; BICU, burn intensive care units; CRP, C‐reactive protein; PCT, Procalcitonin; Pre‐op Hb, pre‐operative hemoglobin; SHAP, the SHapley Additive exPlanations; TBSA, total body surface area.

The flow chart of our research design route is shown in Figure 1. Inclusion criteria: (1) total body surface area (TBSA) ≥ 30%, (2) age > 18 years, (3) from admission to hospital after injury ≤24 h, (4) no clear history of neurological disease, (5) no psychiatric or family history, (6) received surgery, and (7) samples with complete medical records and clinical treatment information. Exclusion criteria: (1) comatose patients, and (2) patients who leave the hospital for various reasons after <7 days of stay.

A total of 47 pre‐operative variables were collected, including patients' personal information (e.g. BMI and education attainment), lifestyle (smoking history and history of alcohol use), pre‐existing diseases (e.g. hypertension, diabetes and heart disease), burn status (e.g. TBSA, burn index and inhalation injury), treatment modality (e.g. mechanical ventilation (MV), number of procedures, number and volume of blood transfusions), treatment environment (e.g. admission to burn intensive care units(BICU), family visits and physical restraint), pre‐operative laboratory parameters (e.g. hemoglobin(Hb), red blood cells (RBC) and white blood cells), Acute Physiology and Chronic Health Evaluation (APACHE II score) and American society of anesthesiologists (ASA) score. All preoperative variables of concern were collected from the electronic case system of the First Affiliated Hospital of Nanchang University and Ganzhou People's Hospital.

### 2.2 Delirium assessment

A variety of delirium assessment tools are currently in clinical use,^20^ of which the Intensive Care Delirium Screening Checklist (ICDSC)^21^ is widely used, particularly in intensive care units (ICU).^13^ ICDSC^21^ has high sensitivity and specificity which helps clinical workers to accurately identify patients with delirium. Some patients with extensive burns require mechanical ventilation, which affects the patient's speech and therefore limits the use of many delirium assessment tools. However, the ICDSC solves this problem. The ICDSC also does not require the additional use of the Richmond Agitation Screening Scale (RASS) and is suitable for the heavy medical environment of the Burns Intensive Care Unit (BICU). In this trial, ICDSC^21^ was used on burn general wards and BICU for extensive burn patients. Patients were evaluated for delirium by four specialized psychiatrists at 12‐h intervals for 5 days after surgery. If the patient was assessed for delirium, the assessment was continued until the patient was in a non‐delirious state. The psychiatrists involved in the delirium assessment were not aware of either the variables included in the study or the study objectives.

### 2.3 Statistical analysis

All data analysis in this study was completed using Python and IBM SPSS 26.0. To avoid overfitting and to obtain more accurate prediction models, an adequate sample size for building prediction models is required. We use a sample size calculated^22^ as *n* = ${\left(\frac{1.96}{\delta }\right)}^{2}\emptyset \left(1-\emptyset \right),\emptyset$ is the expected outcome ratio(∅=0.37), δ is the set margin of error (δ=0.05). According to this formula, the minimum sample size for the training set used to develop the model is 358 individuals.

The normality of the distribution of continuous variables was tested using the Shapiro–Wilk test. Normally distributed continuous variables were expressed as mean ± standard deviation (SD) and compared using the independent samples *t*‐test. Skewed continuous variables were expressed as median and interquartile range (IQR) and compared using the Mann–Whitney *U*‐test. Categorical variables are expressed as frequencies and percentages and using chi‐square tests or Fisher's exact probability tests. This study used multifactorial logistic regression to identify the variables included in the delirium prediction model. In order to achieve the best prediction, nine models were built for this experiment, including an eXtreme Gradient Boosting (XGBoost), a Logistic Regression (LR), a Light Gradient Boosting Machine (LightGBM), a Random Forest (RF), an Adaptive Boosting (AdaBoost), a GaussianNB (GNB), a ComplementNB (CNB), a Multiple‐layers Perceptron (MLP), and a support vector machine (SVM) classifier.

Cross‐validation can better evaluate model performance by averaging multiple experimental indexes. Therefore, the tenfold cross‐validation method was used to validate the models. Filling in missing data: If the percentage of missing values for a variable was higher than 20%, it was excluded from the final complete data set, and if that percentage was lower than 20%, random forest approach was used to fill in the data.

To avoid collinearity between variables and thus affecting the performance of the prediction model, the variables planned for modeling were subjected to multicollinearity and correlation analysis before modeling. Calibration and discriminative were used to test the predictive capability of the predictive models. Decision curve analysis (DCA) assessed the clinical utility of the models. The area under the receiver operating characteristic curve (AUROC) was identified as a measure of discrimination. The calibration plot is used to evaluate the accuracy of the prediction models. The cut‐off value was derived from the Youden index which is (sensitivity + specificity − 1). The best model is selected by comparing the performance between the models. Then the SHapley Additive exPlanations (SHAP) was chosen to explain the best one. Finally, the best model was visualized and then used for external validation. *p* < 0.05 indicated that the difference is statistically significant.

## 3 RESULTS

### 3.1 Participant characteristics

A total of 518 patients participated in the study. Of these, 146 (28.19%) were female and 372 (71.81%) were male. A total of 191 patients developed delirium, and the incidence of delirium was approximately 36.87%. The study variables C‐reactive protein (CRP) and procalcitonin (PCT) were missing >20%, so these two variables were excluded. The missing fraction of preoperative glucose and preoperative albumin accounted for 3.09% and 1.35% of the total data, respectively, and the random forest approach was used to filling the data. The dataset was randomly divided into a training set, a validation set and a testing set. Data from the training set and validation set (*n* = 414, 80% of dataset) are used to build the model. The data from the testing set (*n* = 104, 20% of dataset) is used to further validate the model. Patients were divided into a delirium group (*n* = 191) and a non‐delirium group (*n* = 327) based on the presence or absence of delirium. There were no statistically significant differences in patient characteristics and preoperative variables in the training and testing sets (Table 1).

**TABLE 1:** Demographic characteristics and preoperative variables of patients with extensive burns in the training, validation and test sets.

Variable	All (*n* = 518)	Training and validation set (*n* = 414)	Testing set (*n* = 104)	*p*‐Value
Age, median (IQR)	47.00 (36.00, 54.00)	48.00 (37.00, 54.00)	47.00 (32.00, 55.00)	0.668
Sex				
Male, *n* (%)	372 (71.81)	301 (72.71)	71 (68.27)	0.369
Female, *n* (%)	146 (28.19)	113 (27.30)	33 (31.73)	
BMI, median (IQR)	22.31 (20.76, 24.49)	22.31 (20.66, 24.39)	22.53 (20.83, 24.61)	0.678
Education attainment, *n* (%)				
Lower secondary and below education	378 (72.97)	305 (73.67)	73 (70.19)	0.075
Secondary education	98 (18.92)	81 (19.57)	17 (16.35)	
University education	42 (8.11)	28 (6.76)	14 (13.46)	
APACHE II score, median (IQR)	8.00 (5.00, 10.00)	8.00 (5.00, 10.00)	8.00 (4.00, 11.00)	0.866
ASA degree, median (IQR)	3.00 (3.00, 4.00)	3.00 (3.00, 4.00)	3.00 (3.00, 4.00)	0.313
Smoking, *n* (%)				
None	369 (71.24)	291 (70.29)	78 (75.00)	0.343
Yes	149 (28.76)	123 (29.71)	26 (25.00)	
Alcohol, *n* (%)				
None	495 (95.56)	395 (95.41)	100 (96.15)	0.742
Yes	23 (4.44)	19 (4.59)	4 (3.85)	
Hypertension, *n* (%)				
None	458 (88.42)	367 (88.65)	91 (87.50)	0.744
Yes	60 (11.58)	47 (11.35)	13 (12.50)	
Diabetes, *n* (%)				
None	502 (96.91)	400 (96.62)	102 (98.08)	0.442
Yes	16 (3.09)	14 (3.38)	2 (1.92)	
Cardiovascular disease, *n* (%)				
None	511 (98.65)	410 (99.03)	101 (97.12)	0.130
Yes	7 (1.35)	4 (0.97)	3 (2.88)	
Pulmonary disease, *n* (%)				
None	511 (98.65)	407 (98.31)	104 (100.00)	0.182
Yes	7 (1.35)	7 (1.69)	0 (0.00)	
TBSA, median (IQR)	52.00 (40.00, 68.00)	53.00 (40.00, 70.00)	50.00 (40.00, 65.00)	0.491
Burn Index, median (IQR)	36.50 (26.50, 53.00)	36.00 (26.50, 53.00)	37.00 (26.50, 52.00)	0.750
Burns on the neck, *n* (%)				
None	96 (18.53)	76 (18.36)	20 (19.23)	0.498
Yes	422 (81.47)	338 (81.64)	84 (80.77)	
Inhalation injury, *n* (%)				
None	200 (38.61)	164 (39.61)	36 (34.62)	0.349
Yes	318 (61.39)	250 (60.39)	68 (65.38)	
MV, *n* (%)				
None	341 (65.83)	270 (65.22)	71 (68.27)	0.557
Yes	177 (34.17)	144 (34.78)	33 (31.73)	
Length of MV, median (IQR)	0.00 (0.00, 4.00)	0.00 (0.00, 4.00)	0.00 (0.00, 4.00)	0.472
Admission to BICU, *n* (%)				
None	179 (34.56)	146 (35.27)	33 (31.73)	0.498
Yes	339 (65.44)	268 (64.73)	71 (68.27)	
Time in BICU, median (IQR)	4.00 (0.00, 5.00)	4.00 (0.00, 5.00)	4.00 (0.00, 5.00)	0.889
Physical restraint, *n* (%)				
None	362 (69.88)	284 (68.60)	78 (75.00)	0.203
Yes	156 (30.12)	130 (31.40)	26 (25.00)	
Family visitation, *n* (%)				
None	378 (72.97)	301 (72.71)	77 (74.04)	0.784
Yes	140 (27.03)	113 (27.29)	27 (25.96)	
Bacterial infections, *n* (%)				
None	411 (79.34)	323 (78.02)	88 (84.62)	0.137
Yes	107 (20.66)	91 (21.98)	16 (15.38)	
Surgery time, median (IQR)	90.00 (65.00, 120.00)	88.00 (65.00, 120.00)	90.00 (70.00, 120.00)	0.972
Blood transfusion volume, median (IQR)	3000 (1600, 4900)	3000 (1650, 4900)	3100 (1400, 4900)	0.667
Blood transfusion number, median (IQR)	4.00 (3.00, 6.00)	4.00 (3.00, 6.00)	4.00 (3.00, 6.00)	0.416
Plasma transfusion, median (IQR)	2450 (1200, 4250)	2450 (1200, 4250)	2550 (1200, 4000)	0.740
RCS transfusion, median (IQR)	400 (300, 800)	400 (300, 800)	400 (300, 600)	0.345
PLT transfusion, median (IQR)	0 (0, 0)	0 (0, 0)	0 (0, 0)	0.909
Pre‐op WBC, median (IQR)	9.45 (6.42, 13.21)	9.08 (6.30, 13.04)	10.42 (7.08, 13.82)	0.065
Pre‐op RBC, median (IQR)	4.03 (3.51, 4.56)	4.02 (3.51, 4.55)	4.04 (3.66, 4.57)	0.528
Pre‐op Hb, median (IQR)	122 (103, 137)	121 (103, 137)	124 (106, 137)	0.631
Pre‐op PLT, median (IQR)	133 (92, 185)	129 (90, 178)	142 (98, 204)	0.082
Pre‐op LY, median (IQR)	1.01 (0.72, 1.40)	1.01 (0.70, 1.38)	1.00 (0.75, 1.43)	0.699
Pre‐op LY%, median (IQR)	11.20 (7.80, 15.30)	11.20 (7.90, 15.30)	10.60 (7.60, 15.10)	0.684
Pre‐op NE, median (IQR)	7.24 (4.82, 10.56)	7.10 (4.71, 10.47)	8.36 (5.58, 11.05)	0.069
Pre‐op NE%, median (IQR)	78.60 (73.60, 83.90)	78.60 (73.40, 83.20)	79.30 (74.70, 85.60)	0.113
Pre‐op ALB, median (IQR)	28.50 (25.30, 31.40)	28.30 (25.20, 31.40)	28.90 (26.29, 31.50)	0.333
Pre‐op GLU, median (IQR)	6.84 (5.90, 8.14)	6.95 (5.90, 8.47)	6.64 (5.84, 7.58)	0.071
Pre‐op ALT, median (IQR)	18.00 (13.00, 28.00)	18.00 (13.00, 28.00)	17.00 (13.00, 28.00)	0.841
Pre‐op AST, median (IQR)	27.20 (20.00, 42.00)	27.00 (20.00, 42.00)	28.00 (21.00, 41.00)	0.867
Pre‐op BUN, median (IQR)	5.00 (3.60, 6.90)	5.00 (3.60, 7.10)	5.00 (3.50, 6.10)	0.219
Pre‐op Cr, median (IQR)	63.20 (51.40, 74.70)	63.20 (51.60, 74.70)	63.40 (51.10, 75.90)	0.705
Pre‐op Na+, median (IQR)	136.3 (134.0, 139.0)	136.5 (134.0, 139.0)	136.0 (134.0, 139.1)	0.813
Pre‐op K+, median (IQR)	3.71 (3.43, 4.05)	3.72 (3.47, 4.09)	3.66 (3.40, 3.95)	0.127
Pre‐op Cl‐, median (IQR)	101.7 (99.0, 104.9)	101.7 (99.0, 104.8)	101.6 (99.0, 104.9)	0.893
Pre‐op Ca+, median (IQR)	2.01 (1.88, 2.11)	2.01 (1.88, 2.11)	2.01 (1.87, 2.11)	0.835

Abbreviations: ALB, albumin; ALT, alanine transaminase; APACHE II, Acute physiology and chronic health evaluation II; ASA, American society of anesthesiologists; AST, glutamic oxalacetic transaminase; BICU, burn intensive care units; Blood transfusion volume = plasma + RCS + PLT; BMI, Body mass index (kg/m^2^); BUN, blood urea nitrogen; Cr, serum creatinine; GLU, glucose; Hb, hemoglobin; IQR, interquartile range; LY, lymphocyte; MV, mechanical ventilation; NE, neutrophilicgranulocyte; PLT, platelet; Pre‐op, Pre‐operative; RBC, red blood cell; RCS, red cell suspension; TBSA, total body surface area; WBC, white blood cell.

### 3.2 Selection of key variables

First, a univariate analysis of 47 variables was performed to identify factors associated with the development of delirium in patients with extensive burns. The results of univariate analysis showed that 31 variables were statistically significant. Multifactorial logistic regression analysis was further performed on these 31 statistically significant variables to identify risk factors for the development of delirium in patients with extensive burns. The results of the multifactorial logistic regression analysis showed that eight risk factors namely physical restraint, diabetes, sex, pre‐op Hb, pre‐op RBC, Apache II score, time in BICU and TBSA were associated with the development of delirium in patients with extensive burns. Subsequently, we found that pre‐op Hb and pre‐op RBC had collinearity by multicollinearity analysis (Figure S1A), and the remaining seven variables after excluding pre‐op RBC did not have collinearity (Figure S1B) and were independent of each other (Figure S2). We ultimately modeled this using the following seven variables: physical restraint, diabetes, sex, pre‐op Hb, Apache II score, time in BICU and TBSA.

### 3.3 Establishment of prediction models

**FIGURE 2:**
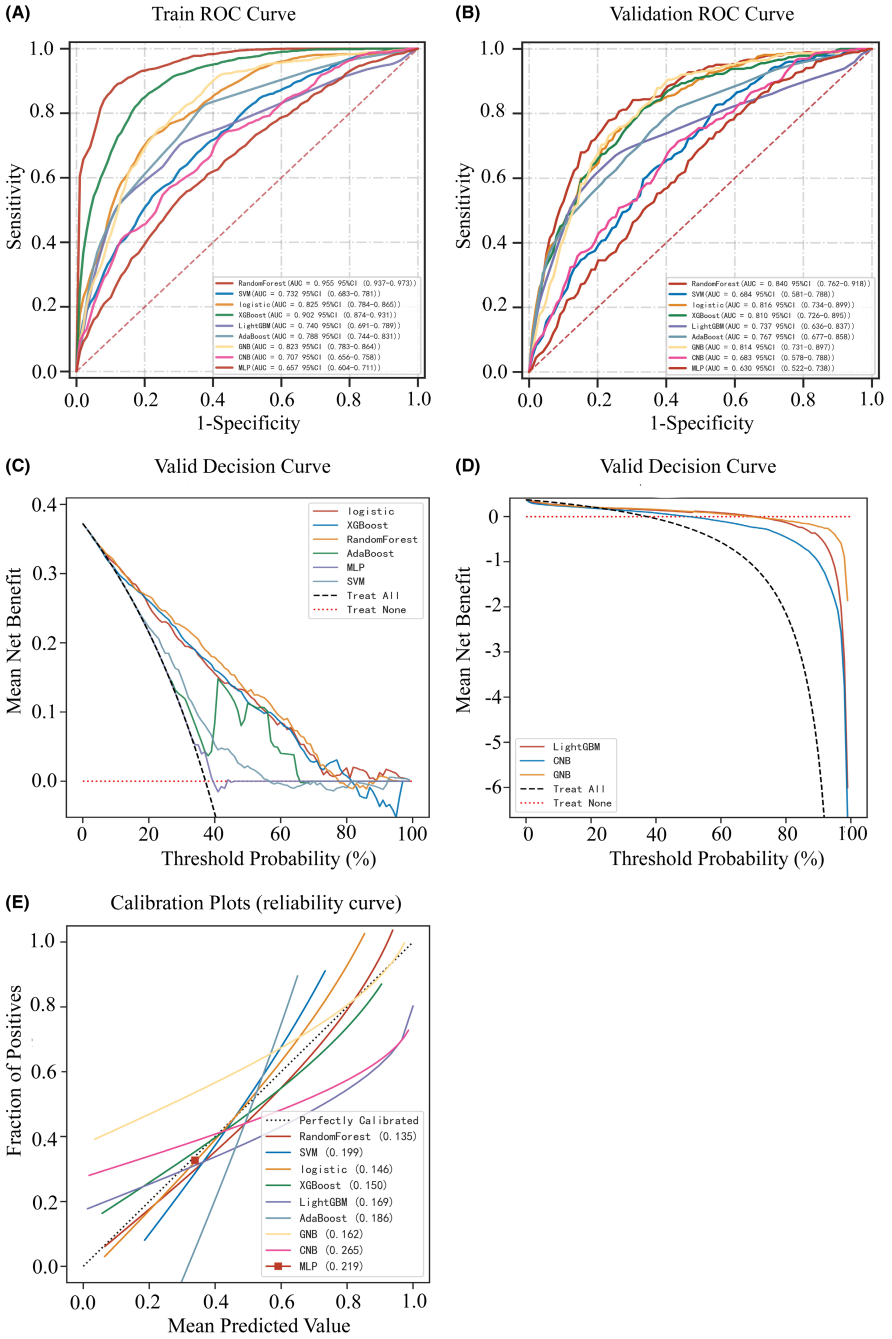
Performance assessment of the models. (A) Receiver operating characteristic curve (ROC) of delirium prediction models in training set. (B) ROC of delirium prediction models in validation set. (C) and (D) Decision curve analysis (DCA) for the nine delirium prediction models in the validation set. (E) Calibration plots of delirium prediction models for patients with extensive burns in the validation set.

Nine prediction models were constructed for delirium prediction in patients with extensive burns using the seven variables mentioned above. AUROC is one of the important indicators used to evaluate delirium prediction models. The ROC curves of the nine prediction models are shown in Figure 2A,B, where the RF prediction model has a better AUROC. Also, we performed a 10‐fold cross‐validation to assess the stability of the prediction models, and the RF prediction model performed better (average AUC = 0.955 ± 0.004). A comparison of the performance of the nine prediction models in the training and validation sets is shown in Table 2 and Table S1, respectively. In addition to the best performance in AUROC, the RF model also performed satisfactorily in terms of sensitivity (0.880), accuracy (0.893) and specificity (0.904).

**TABLE 2:** Performance of models in the training set.

Model	AUC(SD)	Youden Index	Accuracy (SD)	Sensitivity (SD)	Specificity (SD)	PPV (SD)	NPV (SD)	*F* score
XGBoost	0.902 (0.007)	0.667	0.826 (0.011)	0.854 (0.040)	0.813 (0.036)	0.728 (0.027)	0.903 (0.018)	0.785
LR	0.825 (0.008)	0.518	0.763 (0.008)	0.735 (0.031)	0.783 (0.023)	0.663 (0.017)	0.833 (0.013)	0.696
LightGBM	0.740 (0.079)	0.453	0.724 (0.061)	0.648 (0.104)	0.805 (0.084)	0.693 (0.125)	0.738 (0.050)	0.659
RF	0.955 (0.004)	0.784	0.893 (0.005)	0.880 (0.030)	0.904 (0.017)	0.842 (0.019)	0.925 (0.014)	0.860
AdaBoost	0.788 (0.026)	0.484	0.719 (0.037)	0.781 (0.075)	0.703 (0.078)	0.622 (0.073)	0.796 (0.047)	0.685
GNB	0.823 (0.007)	0.527	0.745 (0.021)	0.825 (0.059)	0.702 (0.066)	0.620 (0.035)	0.872 (0.028)	0.705
CNB	0.707 (0.006)	0.332	0.666 (0.029)	0.632 (0.120)	0.690 (0.115)	0.556 (0.063)	0.765 (0.029)	0.578
MLP	0.657 (0.048)	0.262	0.632 (0.046)	0.618 (0.088)	0.644 (0.112)	0.509 (0.047)	0.742 (0.025)	0.552
SVM	0.732 (0.011)	0.351	0.671 (0.030)	0.681 (0.123)	0.670 (0.116)	0.554 (0.044)	0.788 (0.041)	0.601

*Note*: Youden Index = sensitivity + specificity −1. Accuracy = (TP + TN)/(TP + TN + FP + FN). Sensitivity = FP/(FP + TN). Specificity = TN/(FP + TN). PPV = TP/(TP + FP). NPV = TN/(FN + TN). F score = 2/([1/ Recall] + [1/Precision]). Recall = TP/(TP + FN). Specificity = TN/(TN + FP).

Abbreviations: AdaBoost, Adaptive Boosting; AUC, area under the receiver operating characteristic curve; CNB, ComplementNB; FN, false negatives; FP, false positives; GNB, GaussianNB; LightGBM, Light Gradient Boosting Machine; LR, Logistic Regression; MLP, Multiple‐layers Perceptron; NPV, Negative predictive value; PPV, positive predictive value; RF, Random Forest; SVM, support vector machine; TN, true negatives; TP, true positives; XGBoost, eXtreme Gradient Boosting.

**FIGURE 3:**
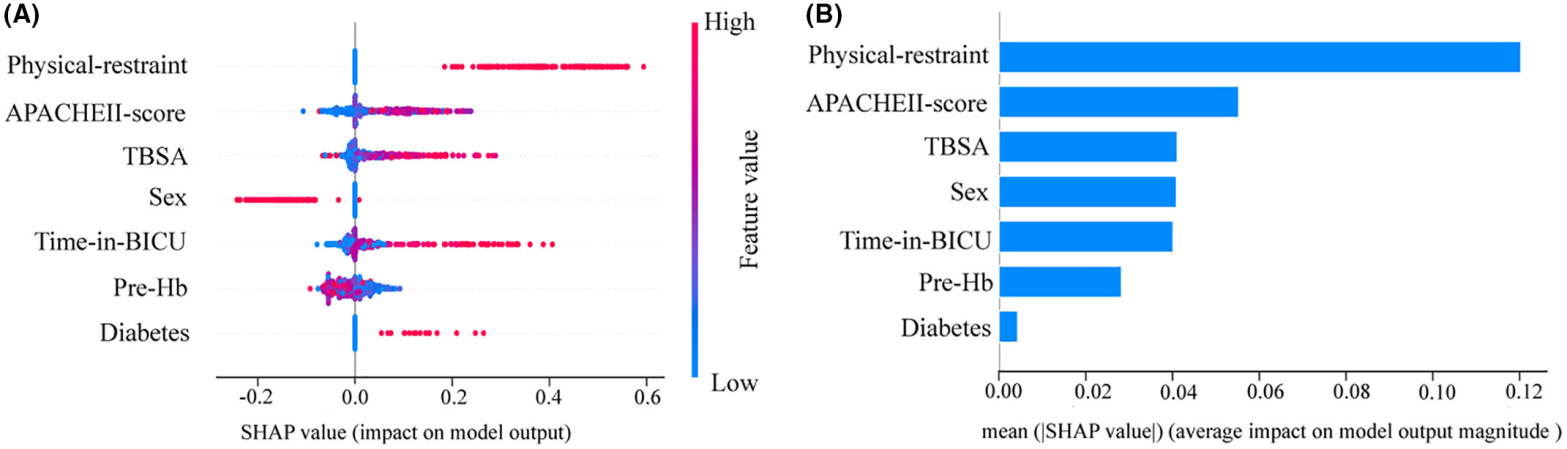
SHAP interprets the RF Predictive model. (A) The SHAP analysis was performed on the RF. Each row of the graph represents a variable and the horizontal coordinate is the SHAP value, which represents the distribution of the effect of the variable on the risk of delirium, with positive values indicating a risk of delirium and negative values indicating no risk of delirium. A point represents a patient, while red represents a higher value and blue represents a lower value. (B) The average of the absolute values of the SHAP values for each variable in the RF is taken as the significance of that variable.

In addition, the RF performs better in terms of DCA results (Figure 2C,D). The calibration curve for The RF is closest to the curve with a slope of 45°, indicating the best accuracy (Figure 2E). In conclusion, we choose the RF prediction model as the best delirium prediction model. The best cut‐off for the RF prediction model was 42.19% according to the Youden index. In the testing set, the AUC value was 82.2%, sensitivity was 89.7%, specificity was 56.9%, accuracy was 72.1%, PPV was 61.9%, NPV was 79.0% and F1 score was 0.733. The SHAP shows the impact of each variable on the predictive power of the model (Figure 3A) and the importance of each variable (Figure 3B).

### 3.4 Validate model performance

**FIGURE 4:**
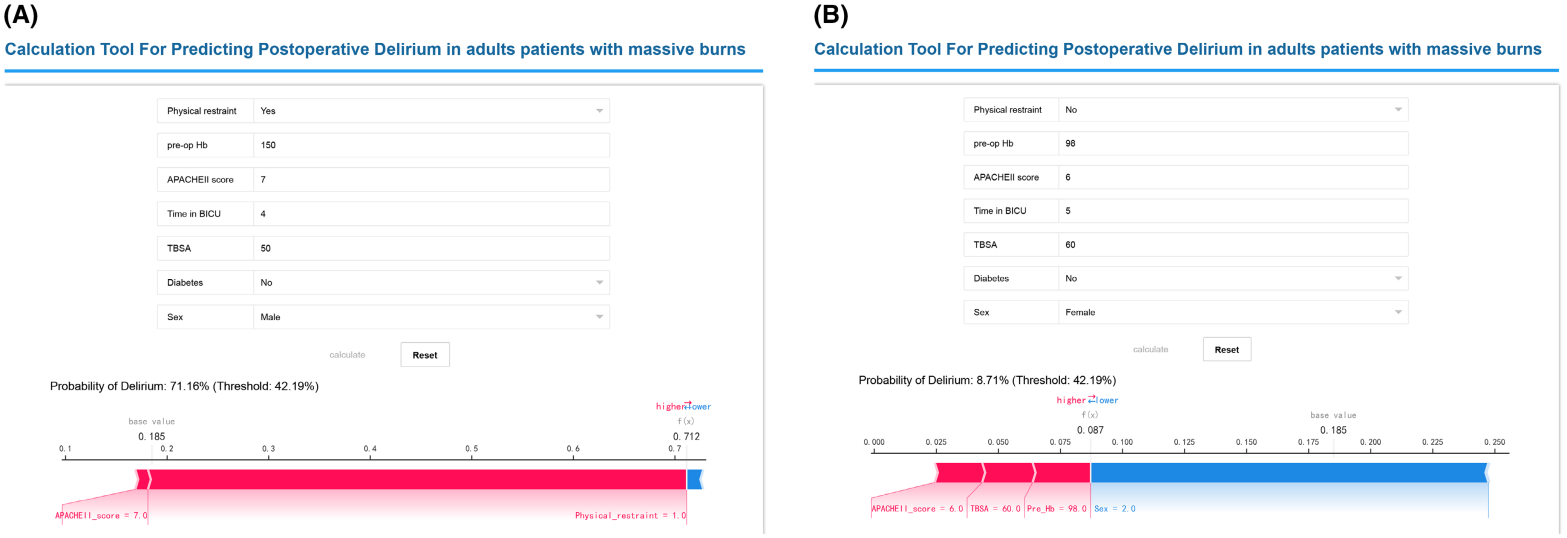
Example of prediction model usage. Enter the preoperative related values to determine the risk of delirium and show the contribution of each value to the prediction. (A) Example 1 high risk of delirium, (B) Example 2 low risk of delirium.

Visualization of the RF prediction model using a web‐based calculator (available at: https://www.xsmartanalysis.com/model/predict/?mid=2127&symbol=1iO16fE64184FN0220eQ). We collected data from 118 patients for external validation of the prediction model. None of the required preoperative variables were missing in the external validation. The external validation dataset contained 96 male patients and 22 female patients. Delirium occurred in 31 (26.27%) of these patients. There were no significant differences in demographic characteristics between delirious and non‐delirious patients. The RF prediction model had an accuracy of 77.12%, a specificity of 80.46%, and a sensitivity of 67.74% in the external validation. A web‐based calculator score > 42.19% indicates that the patient is at risk for delirium. The following preoperative information was entered into the model for one patient: sex male, TBSA of 50%, no diabetes, Apache II score of 7, physical restraint, pre‐op Hb of 150 g/L, and a 4‐day stay in the BICU. The prediction model assessed a score of 71.16%, suggesting to the medical staff that this patient has a high likelihood of delirium and should be given the appropriate interventions (Figure 4A). Preoperative information for another patient was entered into the model: sex was female, TBSA was 60%, no diabetes, Apache II score was 6, no physical restriction, pre‐op Hb was 98 g/L and 5 days in the BICU. The predicted probability of delirium for this patient was 8.71%, indicating a low risk of delirium in this patient (Figure 4B).

## 4 DISCUSSION

There are no models for predicting delirium in patients with extensive burns, and this study is innovative in predicting the risk of delirium in patients with extensive burns using machine learning algorithms. The RF prediction model we developed accurately predicts the probability of postoperative delirium in patients, with satisfactory discrimination and generalization compared to the other eight prediction models.

Delirium is diagnosed mainly by clinical manifestations and adjunctive delirium assessment tools, with the potential for underdiagnosis of hypoactive delirium. Therefore, in recent years, scholars have focused on finding objective indicators that can help diagnose delirium, such as biomarkers^23^ and electroencephalography (EEG).^5^ Multiple biomarkers such as CRP,^24^ Cerebral spinal fluid (CSF)^25^ and plasma tau^26^ have been used in experimental studies, but due to the complexity of biomarker extraction procedures, it is difficult to apply them clinically. Therefore, we considered whether we could analyze the clinical data to seek commonalities to the extent that early prediction and early intervention of burn delirium could be achieved.

Delirium is a complex disease caused by a combination of factors. It is also a common complication in patients with extensive burns, and the incidence of delirium in this study was 36.87%. Targeted interventions for high‐risk patients can be effective in reducing the incidence of delirium.^13^ Preoperative high‐dose glucocorticoids reduce the incidence of delirium in adult patients 4 days after hepatectomy.^27^ Perioperative use of dexmedetomidine is effective in preventing delirium in elderly patients undergoing open esophagectomy.^28^ However, there is a lack of consensus and no guidelines to clearly define patients at ‘high risk’ of delirium.^13^ Therefore, it is important to use delirium prediction models to objectively identify high‐risk patients for clinicians.

There are a variety of methods available for developing predictive models, and using advanced statistical methods such as machine learning to develop predictive models can improve the efficacy of the models.^29^ The formula for prediction model is complex and currently more studies choose to translate the prediction model into a nomogram for clinical use.^30^, ^31^ However, Nomogram requires the user to perform manual calculations based on various information about the patient, which increases the workload of healthcare professionals to some extent, and there is also a risk of calculation errors leading to incorrect assessments. We chose the web‐based calculator to visualize the prediction model and input information about patients with extensive burns for automatic calculation to improve ease‐of‐use.

In addition, we visualize how the model predicts the incidence of delirium in patients with two examples and assess the relative importance of each risk factor acting on the patient. Given the large number of patients with extensive burns each year, these findings can help doctors to better formulate treatment plans and allocate medical resources more appropriately, as well as help patients and their families to understand the possibility of delirium and better understand treatment plans so that they can cooperate more actively with treatment and improve compliance.

Prediction models usually choose indicators that are routinely used in clinical practice to improve the applicability of the prediction model, and there is a possibility of exaggerating the model performance if postoperative indicators are chosen.^18^ In addition, some patients develop delirium a few hours after surgery and the use of intraoperative and postoperative indicators for prediction may delay the implementation of interventions for the patient.

Currently, the inflammatory hypothesis is now considered to be one of the pathogenic mechanisms of delirium^32^ and many studies point to indicators such as CRP^24^ and PCT^33^ as predictors of delirium in surgical patients. However, in screening the variables used to construct the predictive model, we excluded inflammatory mediators. On the one hand, this was because there were too many missing data for CRP and PCT to fill in the data with random forest regression. On the other hand, infections do not always occur in other surgical patients, but they can occur in every patient with extensive burns due to a compromised skin barrier, immune dysfunction and invasion by pathogenic bacteria.^34^ This is why the inflammatory index results are high in patients with extensive burns. Therefore, the differences in these variables were not statistically significant when statistical analyses were conducted and they were not ultimately used in the modeling for this study.

In our study, we developed a delirium prediction model using seven variables: physical restraint, diabetes, sex, pre‐op Hb, Apache II score, time in BICU and TBSA. Of these, the RF was translated into a web‐based calculator for medical staff as the best predictive model, with a score >42.19% indicating that the patient was at greater risk of delirium and should be intervened with. In the training and validation sets, the RF had the largest AUC values of 95.5% and 84% compared to the other eight models, respectively, and had better sensitivity and specificity. In addition, the RF showed good generalization with satisfactory accuracy when externally validated at another large medical center. This confirms the feasibility of extrapolating the predictive model we have developed to clinical use.

Meanwhile, we found that sex was associated with the incidence of delirium, which is consistent with previous findings.^35^, ^36^ Estrogen may be a protective factor for cognitive performance.^37^ In addition, men are more in exposure than women to risk factors that impair cognitive function, such as obstructive sleep apnea^38^ and alcohol use.^39^ On the other hand, different sex may produce different mental disorders under the influence of changes in CRF signaling that occur in the acute stress response.^40^ This study confirms that physical restraint is an independent risk factor for delirium in burn patients, and the SHAP analysis also shows that physical restraint is most strongly associated with delirium in patients with extensive burns. This is consistent with previous studies.^41^, ^42^ It is therefore necessary to use physical restraints with caution and for shorter periods of time.^43^ Several studies exploring risk factors for delirium have shown that diabetes and Apache II score play an important role in predicting the onset of delirium.^44^, ^45^ These studies support the findings of this study. In addition to this, other factors that increased the risk of delirium in patients with extensive burns were identified in this study, including time in BICU and TBSA. The skin is the largest organ of the body and burns result in varying degrees of skin damage,^1^ and TBSA is an important factor used to measure the extent of the condition after a burn injury. The larger the TBSA, the more complex the patient's condition, which often involves multiple surgeries and a high risk of infection,^3^, ^46^ with a consequent increase in the length of stay in the BICU.^47^ In addition, the larger the TBSA, the more complex the use of sedative and analgesic medications.^48^, ^49^ These conditions increase the risk of delirium in patients.

Hb is the protein that transports oxygen within the red blood cells, and the trend in red blood cell and Hb content is consistent. In the study, the results of the multifactorial logistic regression analysis showed that both pre‐op Hb and pre‐op RBC were associated with the development of delirium in the patients, and we found that this result could be due to the multicollinearity of these two factors through the analysis of multicollinearity. At the same time, previous studies have shown that Hb^50^, ^51^ and delirium are more strongly correlated than RBC.^52^ Therefore, to avoid affecting the performance of the model and to improve the accuracy of the prediction model, we finally chose to include pre‐op Hb in the prediction model and exclude pre‐op RBC. Hb may contribute to delirium by affecting the oxygen saturation of brain tissue.^53^ Low Hb may also have a negative impact on brain white matter integrity^54^ and cognitive function.^55^ Therefore, the patient's blood count should be reviewed regularly during treatment to correct the low Hb according to the patient's actual condition.

The purpose of this study was to develop a well‐performing delirium prediction model to predict the risk of delirium in patients with extensive burns in order to help healthcare professionals identify patients at high risk of delirium early and provide non‐pharmacological interventions, such as ABCDEF bundle^56^ to reduce the exposure of high‐risk patients to risk factors. This can reduce the incidence of delirium in patients with extensive burns and has positive implications for improving prognosis, protecting patients' cognitive abilities, reducing the financial burden on families and saving medical resources. The use of machine learning to build disease prediction models can help clinicians become more aware of the importance of various risk factors acting on the organism, as well as pushing medical practitioners to explore the underlying pathophysiology of disease through risk factors. Therefore, as future work, we plan to optimize our predictive model and embed it in the clinical case system to provide a simpler and more practical tool for medical practitioners.

It is worth mentioning some of the contributions made by our study. The incidence of delirium in patients with extensive burns is high, but has rarely been studied. We explored the risk factors for the development of delirium in patients with extensive burns through a retrospective analysis. Also, as far as we know, previous researchers have not developed and validated predictive models for the onset of delirium in patients with extensive burns, and we have taken the first step in this exploration. This has also served to increase the amount of literature on delirium prediction based on machine learning. Compared with the existing literatures in this research area, our study has breakthroughs in terms of sample size, model selection, and model visualization methods. For example, our sample size was larger compared to previous studies (*N* = 518 compared to *N* = 308–380).^57^, ^58^, ^59^ Meanwhile, many previous studies only build a single prediction model, while this study builds nine prediction models to select the best performing prediction model by comparison.^57^, ^58^, ^59^ In addition, unlike other studies that transformed the model into a nomogram, we opted for a simpler and faster online tool.^31^ Currently, most prediction models are built and only internally validated, the fact that external validation was carried out in this study is another major advantage. In external validation, the model performed well with satisfactory accuracy. This provides strong evidence for the extrapolation of the predictive model and suggests to us that the predictive model is feasible for practical application in the clinic. Nevertheless, our study has shortcomings: (1) We are a retrospective study and there is a problem of missing data. Filling in missing data is a complex problem, and different approaches to dealing with missing data have different degrees of impact on prediction model performance. However, our study chose the random forest approach to fill in the missing data and minimize the interpolation error^60^; (2) all the data used to develop the prediction model in this study were from the same large burn center, and we only performed external validation at one hospital. Performing multi‐center external validation may be more helpful in extrapolating the prediction model; (3) there are relatively small numbers of patients with large burns, and although we have an adequate sample size, a larger sample size would be more helpful in optimizing the model. Meanwhile, we would like to build ensemble models in the future based on obtaining larger sample sizes. The model is also interpreted in an easy‐to‐understand manner so as to improve the delirium prediction model. So, we are now embarking on a multi‐canter, larger sample study to refine this prediction model and further improve its generalization.

## 5 CONCLUSION

In this study, we used machine learning algorithms incorporating seven preoperative variables to build nine predictive models of delirium in patients with extensive burns and compared the efficacy of the models using AUROC, DCA and the calibration plot to finally select and visualize the best performing predictive model. It is the first model to achieve individualized prediction of delirium in patients with extensive burns, and is simple to use with good performance and generalization capabilities. We recommend that the model be used routinely to predict the risk of delirium in patients with extensive burns and that aggressive interventions be made for high‐risk patients to help reduce the incidence of delirium, thereby reducing the physical, psychological and financial burden on patients, as well as reducing the workload of doctors and nurses and allowing for a more rational allocation of healthcare resources.

## FUNDING INFORMATION

This study was supported by Science and Technology Research Project of Education Department of Jiangxi Province [grant numbers GJJ2200141].

## CONFLICT OF INTEREST STATEMENT

The authors declare no conflicts of interest.

## Supporting information


Figure S1.

Figure S2.

Table S1.

## Data Availability

The data that support the findings of this study are available from the corresponding author upon reasonable request.
