# Supplementary figures and images for: Machine learning for prediction of delirium in patients with extensive burns after surgery
Source: CNS Neurosci Ther. 2023 Apr 30;29(10):2986–97. doi: 10.1111/cns.14237 (PMC10493655; doi:10.1111/cns.14237)

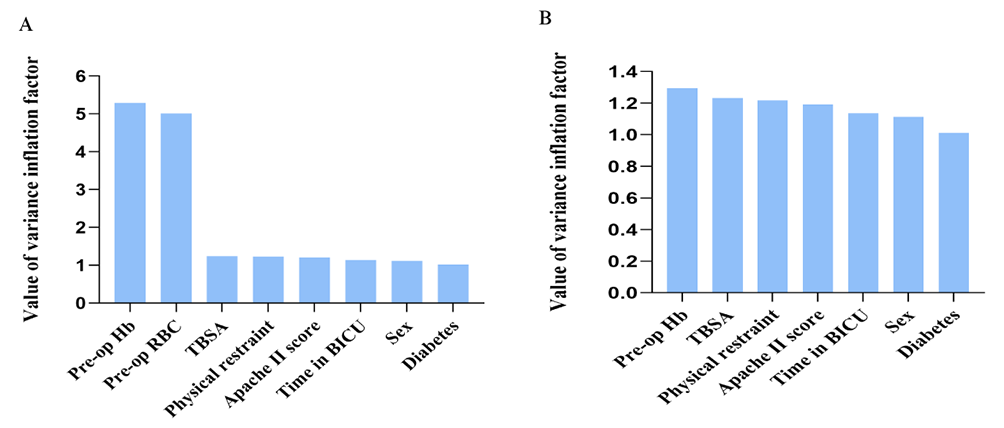

Supplement: Supplementary file 1 — Figure S1. [file CNS-29-2986-s001.zip › CNS_14237_Figure S1.tif]

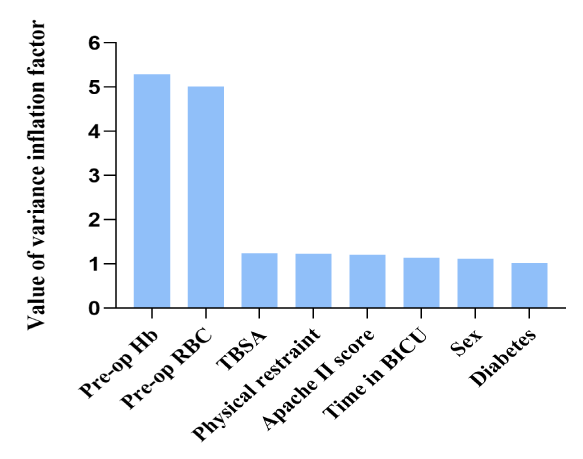

Supplement: Supplementary file 1 — Figure S1. [file CNS-29-2986-s001.zip › CNS_14237_Figure S1A.tif]

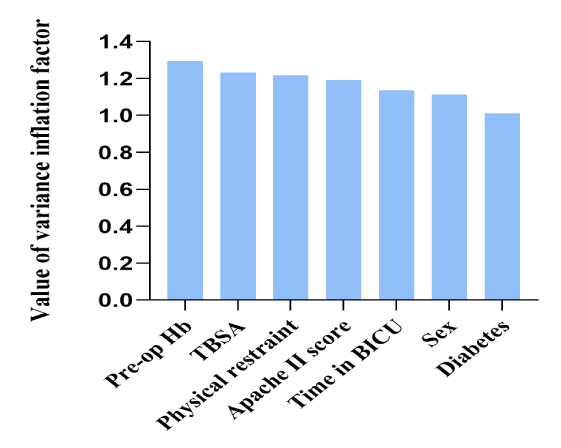

Supplement: Supplementary file 1 — Figure S1. [file CNS-29-2986-s001.zip › CNS_14237_Figure S1B.tif]

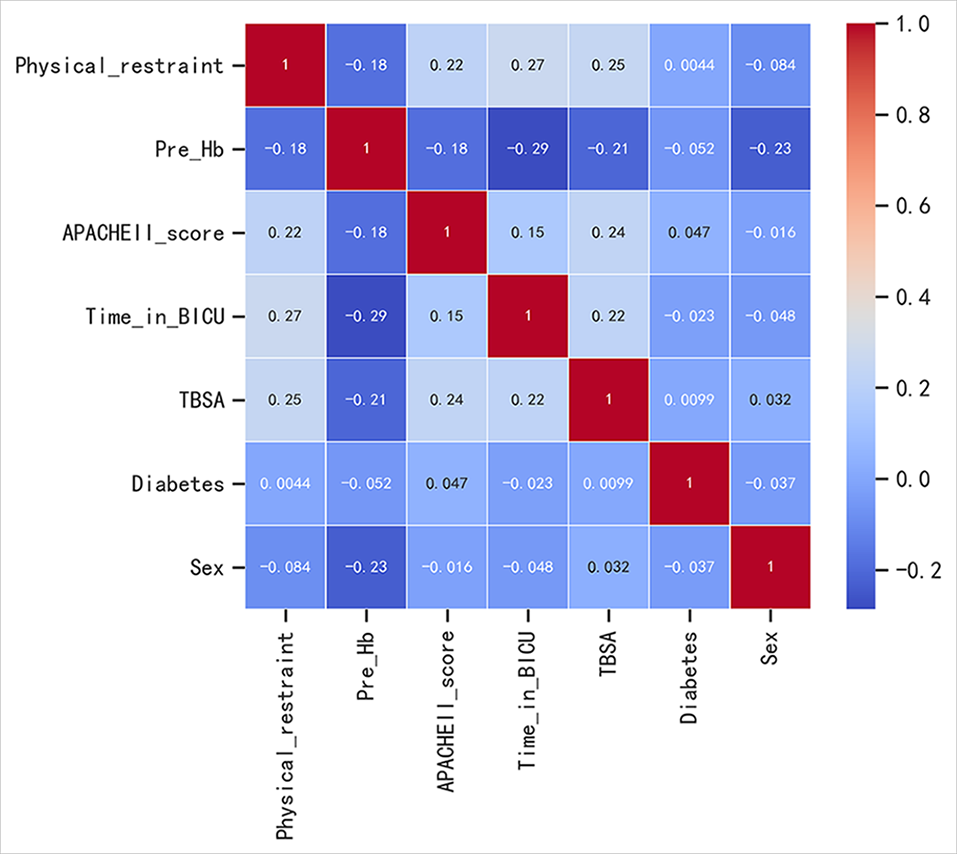

Supplement: Supplementary file 2 — Figure S2. [file CNS-29-2986-s003.tif]
