# Supplementary material for: Machine learning for prediction of delirium in patients with extensive burns after surgery
Source: CNS Neurosci Ther. 2023 Apr 30;29(10):2986–97. doi: 10.1111/cns.14237 (PMC10493655; doi:10.1111/cns.14237)
Supplement: Supplementary file 3 — Table S1. [file CNS-29-2986-s002.doc]

**Table S1.** Performance of models in the validation set.

| **Model** | **AUC(SD)** | **Youden**  **Index** | **Accuracy (SD)** | **Sensitivity (SD)** | **Specificity (SD)** | **PPV (SD)** | **NPV (SD)** | **F1 score** |
| --- | --- | --- | --- | --- | --- | --- | --- | --- |
| **XGBoost** | 0.810(0.039) | 0.532 | 0.735(0.032) | 0.804(0.115) | 0.728(0.114) | 0.629(0.059) | 0.819(0.046) | 0.700 |
| **LR** | 0.816(0.030) | 0.527 | 0.751(0.029) | 0.755(0.062) | 0.772(0.077) | 0.659(0.060) | 0.817(0.038) | 0.701 |
| **LightGBM** | 0.737(0.077) | 0.464 | 0.731(0.057) | 0.628(0.123) | 0.836(0.078) | 0.723(0.112) | 0.736(0.060) | 0.663 |
| **RF** | 0.840(0.032) | 0.590 | 0.775(0.037) | 0.802(0.052) | 0.788(0.052) | 0.703(0.052) | 0.820(0.043) | 0.748 |
| **AdaBoost** | 0.767(0.051) | 0.465 | 0.695(0.050) | 0.728(0.128) | 0.737(0.130) | 0.593(0.086) | 0.768(0.051) | 0.642 |
| **GBN** | 0.814(0.027) | 0.558 | 0.711(0.021) | 0.860(0.073) | 0.698(0.085) | 0.589(0.052) | 0.841(0.061) | 0.697 |
| **CNB** | 0.683(0.024) | 0.337 | 0.617(0.031) | 0.715(0.181) | 0.622(0.191) | 0.487(0.040) | 0.719(0.044) | 0.569 |
| **MLP** | 0.630(0.052) | 0.265 | 0.588(0.045) | 0.775(0.129) | 0.490(0.147) | 0.458(0.042) | 0.699(0.063) | 0.572 |
| **SVM** | 0.684(0.045) | 0.345 | 0.620(0.040) | 0.752(0.153) | 0.593(0.160) | 0.501(0.085) | 0.732(0.054) | 0.593 |

Youden Index= sensitivity + specificity -1. Accuracy = (TP + TN)/(TP + TN + FP + FN). Sensitivity =FP / (FP + TN). Specificity= TN / (FP + TN). PPV=TP / (TP + FP). NPV= TN / (FN + TN). F1 score = 2/([1/ Recall] + [1/Precision]). Recall = TP/(TP + FN). Specificity = TN/(TN + FP). FN, false negatives; FP, false positives; TN, true negatives; TP, true positives; AUC, area under the receiver operating characteristic curve; PPV, positive predictive value; NPV, Negative predictive value; XGBoost, eXtreme Gradient Boosting; LightGBM, Light Gradient Boosting Machine; LR, Logistic Regression; RF, Random Forest; AdaBoost, Adaptive Boosting; GNB, GaussianNB; CNB, ComplementNB; MLP, Multiple-layers Perceptron; SVM, support vector machine.
